# Hippocampus Is Place of Interaction between Unconscious and Conscious Memories

**DOI:** 10.1371/journal.pone.0122459

**Published:** 2015-03-31

**Authors:** Marc Alain Züst, Patrizio Colella, Thomas Peter Reber, Patrik Vuilleumier, Martinus Hauf, Simon Ruch, Katharina Henke

**Affiliations:** 1 Department of Psychology, Division of Experimental Psychology and Neuropsychology, University of Bern, Bern, Switzerland; 2 Center for Cognition, Learning and Memory, University of Bern, Bern, Switzerland; 3 Department of Neurosciences and Clinical Neurology, University of Geneva, Geneva, Switzerland; 4 Institute of Diagnostic and Interventional Neuroradiology, University Hospital Bern, Bern, Switzerland; French National Centre for Scientific Research, FRANCE

## Abstract

Recent evidence suggests that humans can form and later retrieve new semantic relations unconsciously by way of hippocampus—the key structure also recruited for conscious relational (episodic) memory. If the hippocampus subserves both conscious and unconscious relational encoding/retrieval, one would expect the hippocampus to be place of unconscious-conscious interactions during memory retrieval. We tested this hypothesis in an fMRI experiment probing the interaction between the unconscious and conscious retrieval of face-associated information. For the establishment of unconscious relational memories, we presented subliminal (masked) combinations of unfamiliar faces and written occupations (“actor” or “politician”). At test, we presented the former subliminal faces, but now supraliminally, as cues for the reactivation of the unconsciously associated occupations. We hypothesized that unconscious reactivation of the associated occupation—actor or politician—would facilitate or inhibit the subsequent conscious retrieval of a celebrity’s occupation, which was also actor or politician. Depending on whether the reactivated unconscious occupation was congruent or incongruent to the celebrity’s occupation, we expected either quicker or delayed conscious retrieval process. Conscious retrieval was quicker in the congruent relative to a neutral baseline condition but not delayed in the incongruent condition. fMRI data collected during subliminal face-occupation encoding confirmed previous evidence that the hippocampus was interacting with neocortical storage sites of semantic knowledge to support relational encoding. fMRI data collected at test revealed that the facilitated conscious retrieval was paralleled by deactivations in the hippocampus and neocortical storage sites of semantic knowledge. We assume that the unconscious reactivation has pre-activated overlapping relational representations in the hippocampus reducing the neural effort for conscious retrieval. This finding supports the notion of synergistic interactions between conscious and unconscious relational memories in a common, cohesive hippocampal-neocortical memory space.

## Introduction

Episodic memory is a class of declarative memory thought to depend on consciousness of encoding and retrieval [1–3]. The hippocampus is the neuroanatomical hub governing the encoding and retrieval of episodic memories. Damage to the hippocampal-anterior thalamic axis produces severe impairments of episodic memory, but leaves unconscious forms of memory such as skill-learning or priming intact because these forms of memory depend on extrahippocampal structures [1–3].

Recent evidence suggests, however, that episodic memory formation and retrieval is possible even without conscious awareness of encoding and retrieval, and that both encoding and retrieval depend on the hippocampal-anterior thalamic axis [4]. These findings question classic notions of separate memory systems [1–3] and support the processing-based memory model [5] that distinguishes memory systems based on processing modes rather than consciousness. The processing-based memory model distinguishes between memory systems with respect to 3 variables: speed of encoding (rapid versus slow), nature of representation (flexible versus rigid), and memory content (single items versus associations). This model hypothesizes the existence of both a conscious and unconscious form of episodic memory with both forms depending on the hippocampal anterior-thalamic axis. Consciousness, therefore, is not prerequisite for relational encoding and retrieval but rather an independent factor that serves the strengthening of hippocampal memory representations [4].

If episodes can be encoded with and without consciousness by way of the hippocampal anterior-thalamic axis and related cortices [6–8], the organization of consciously and unconsciously acquired information in a single, cohesive hippocampal memory space is economically and evolutionarily sensible. Linked episodic knowledge—conscious and unconscious—informs and guides us better through life than episodic knowledge that is stored separated according to levels of representation from conscious to unconscious. Episodic memories are dynamic and subject to transformation from conscious to unconscious and vice versa. Consider an unconscious memory trace that suddenly “pops” into consciousness, or implicit knowledge of a hidden sequence in a serial reaction time task [9], or a rule in the number reduction task [10,11] that become consciously accessible following sleep. Conversely, memory traces can also get purged from conscious access dropping to a pre-conscious representation [12]. Consciously encoded memories can also become inaccessible when one is instructed to forget them [13]. In all of these cases, a cohesive memory space provides for a stable organizational structure of memory that allows for shifts in the level of representation from unconscious to conscious and vice versa. Such representational shifts appear more difficult if one assumes a strict division between memory systems based on conscious access.

If conscious and unconscious episodic memories are both accommodated by the hippocampal memory system, they can be expected to interact both synergistically and competitively. For example, the activation of *unconscious* memories may facilitate the subsequent formation and retrieval of content-congruent *conscious* memories through activation of nearby or overlapping neural assemblies. We tested this hypothesis using functional magnetic resonance imaging (fMRI). In particular, the hippocampus was hypothesized to be place of interactions between unconscious and conscious retrieval processes.

**Fig 1:**
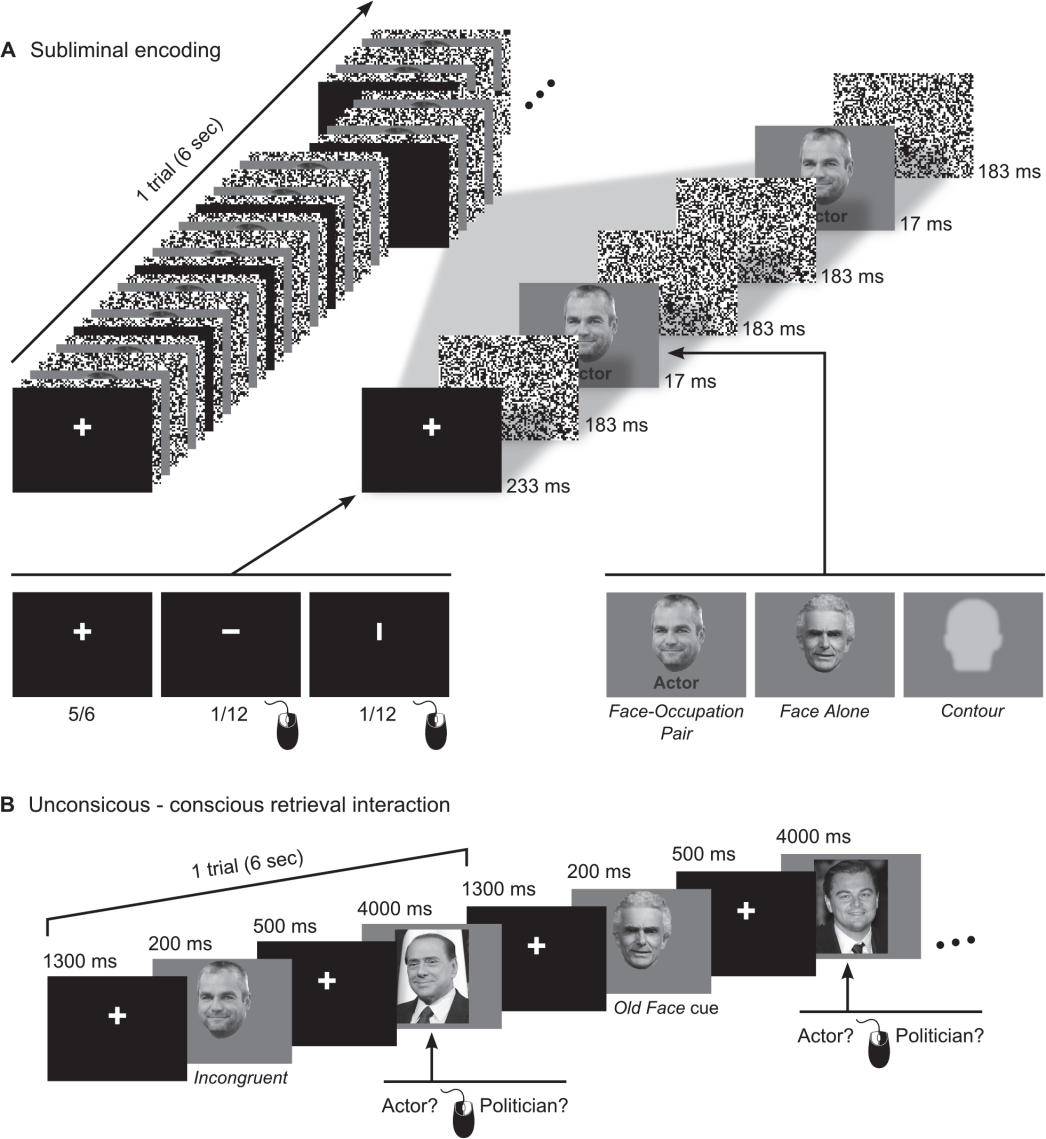
Experimental design. **A:** Attention task during subliminal encoding. Participants saw a flickering stream of black-and-white pixel masks. Subliminal stimuli were presented between masks. The top left depicts one encoding trial containing twelve repetitions of one subliminal stimulus. Four encoding trials constitute a condition block in this fMRI design. On the top right, a section of an encoding trial is highlighted with indicated presentation durations. To the lower left, the used fixation screens are displayed with their respective frequencies of appearance. Each encoding trial contained one response slide (either a vertical or horizontal line segment). To the lower right, we display the three stimulus categories that belong to the three experimental encoding conditions (from left to right): *Face-Occupation Pairs* for associative encoding, *Faces Alone* for single item encoding (non-associative baseline) and *Contour* for a non-encoding baseline (not discussed in this paper). Portraits belong to the FERET database *[15]*. **B:** Unconscious-conscious retrieval interaction with indicated presentation durations. A former subliminal face is briefly presented to cue the unconscious reactivation of previously formed face-occupation association. Next, a portrait of a celebrity comes up for the conscious retrieval of the celebrity’s occupation (actor or politician) Participants were required to recognise the famous person and to indicate his occupation by button press. Each condition block contained four trials. Fig 1B illustrates a trial of the associative retrieval condition *Incongruent* and a trial of the *Old Faces* baseline condition, where no unconscious-conscious interaction was possible. Celebrities’ portraits were taken from Wikimedia Commons (http://commons.wikimedia.org/wiki/Main_Page). Berlusconi: public domain; DiCaprio: Siebbi (http://www.ipernity.com/home/siebbi).

Participants were first presented with subliminal combinations of unfamiliar faces and occupations (face plus the label “actor” or face plus the label “politician”) for unconscious relational encoding. Due to the relational nature of unconscious memory formation, we expected the hippocampus to be activated during unconscious encoding. Following the subliminal presentation of face-occupation combinations, an unconscious-conscious retrieval interaction test was given. We studied whether the unconscious reactivation of the earlier formed face-occupation association would facilitate or inhibit the conscious retrieval of a stored association between a celebrity’s face and his occupation, namely actor or politician. We used portraits of famous actors and politicians as cues for the conscious retrieval of occupations. This relational retrieval draws on both the episodic and the semantic (facts) memory system depending on the experience that the young participants in our study had with movies and political shows/news [14]. Each test trial included the brief but visible presentation of a former subliminal face, stripped off its occupation label, followed by the presentation of the portrait of a celebrity. Participants were instructed to react to the famous face by deciding whether the depicted person was an actor or a politician (Fig 1).

We expected that the former subliminal face’s occupation (actor or politician) would be reactivated unconsciously and would facilitate or inhibit the conscious retrieval of the celebrity’s occupation (actor or politician) depending on whether the two faces share occupations or not. A facilitating interaction may render the conscious retrieval more efficient reducing neural activation and reaction times. Conversely, an inhibitory interaction may increase neural activation and reaction times. These two conditions were contrasted to a baseline condition that provided for unconscious face encoding and retrieval without relational demands and hence was neutral regarding occupational categorization.

The hypothesized neural basis of unconscious-conscious interactions comprises the neocortical storage sites of occupations, namely the lateral and polar temporal cortex [16,17], as well as the hippocampal-anterior thalamic axis. Evidence in favour of a common memory space for both unconscious and conscious relational memories would speak to a common memory system for conscious and unconscious relational (i.e., episodic) memories. Such evidence would challenge the traditional segmentation of memory systems according to consciousness [1–3] and would support the processing-based memory model [5].

## Methods

### Participants

Forty-two healthy male volunteers (age 19–32 years; *M* ± *SD* = 23.86 ± 3.02) participated in the experiment. They denied previous or current neurological or psychiatric disorders and drug abuse. Each participant fulfilled inclusion criteria and no exclusion criteria for MRI. All participants were right handed [18] and had normal or corrected-to-normal vision. Participants gave semi-informed consent. They were not informed of subliminal presentations until debriefing following the fMRI experiment. This study was approved by the local ethics committee for human studies (Kantonale Ethikkommission Bern).

Two participants were excluded from data analysis due to their insufficient acquaintance with the famous faces used in the experiment. Seven further participants were excluded because of their above-chance performance on the awareness tests (cf. section 0). Accordingly, thirty-three participants entered data analyses.

### Material

#### Stimuli

For subliminal encoding, 216 frontal portraits of unknown male faces were retrieved from the internet using Google (http://images.google.com) and the FERET database [15]. The portraits were converted to grayscale, realigned and contrast-reduced. They were then validated concerning their prototypicality for the occupations “actor” and “politician” by 32 (separate) students, who agreed to evaluate the faces in an online experiment using a forced-choice task. The 148 least prototypical portraits were used as a stimulus pool for the experiment. Forty-eight portraits were used in the main experiment and the remaining 100 portraits were used in the awareness tests. The assignment of faces to these two lists was randomized for each participant. A contour of a human head was reduced in contrast and blurred to be used in a baseline condition in the subliminal encoding part of the fMRI experiment

For the test of an unconscious-conscious interaction during retrieval we collected frontal portraits of 32 famous male actors and 32 famous male politicians from the internet. These portraits were also grayscaled and realigned but not contrast-reduced because they were not used for subliminal presentation.

#### Setup

Stimuli were presented with a Benq© WXGA SP830 DLP video projector using a resolution of 1024 × 768 pixels and a screen refresh rate of 60 Hz. Stimuli were projected onto a backlit screen with a viewing angle of 16° width and 9° height. Stimulus presentation was programmed with the software Presentation Version 11.3 (Neurobehavioral Systems, http://www.neurobs.com). Participants responded by key press on a Lumina Response Pad LU400-Pair by Cedrus (www.cedrus.com/lumina) while lying in the MR Scanner.

### Experimental procedure

The experiment was carried out in a dimmed MRI chamber. The study encompassed the following phases in this order: 1) a conscious memory task was given to establish a task-set that prepares participants for unconscious associative encoding, 2) the fMRI experiment encompassing subliminal encoding and a test of unconscious-conscious interaction during retrieval, 3) a test of stimulus awareness, and 4) the explicit identification of famous faces. Phases 1 through 3 were carried out while the participants where situated inside the MR scanner.

The experiment was designed to suit an fMRI block design with alternating condition blocks. There were two fMRI time-series, one for subliminal encoding and the other for the interaction test. All condition blocks took 24 seconds and contained four trials spanning 6 seconds each. The assignment of stimuli to conditions and of occupations to faces was pseudo-randomized. Condition blocks alternated regularly in a fixed order. The starter block varied between participants to distribute over experimental conditions certain psychological dispositions such as stress or fatigue and the pervasive scanner drift.

#### Subliminal encoding

We used our established presentation protocol with subliminal stimuli embedded in an attention task [7] (Fig 1A). Initially, a fixation cross (F) was presented for 233 ms. Four noise masks (M) were then presented for 183 ms each. Between the noise masks, stimuli (S) were presented subliminally for 17 ms. Stimuli were either *Face-Occupation Pairs*, *Faces Alone* (= non-associative baseline) or *Contour* (not discussed in this study). The noise masks served as forward- and backward masks [7,19]. One trial took six seconds, consisted of 6 sub-trials and ran down in the following order: 6 × (F-M-S-M-M-S-M). This resulted in 12 consecutive subliminal presentations of a stimulus. In each trial, one of the six fixation crosses was replaced by either a horizontal or a vertical line segment. These replacements had to be acknowledged by participants with key press responses. This attention task ensured that participants’ attention remained focused on the centre of the screen throughout the task.

Subliminal encoding was implemented as a block design with three alternating conditions, namely *Face-Occupation Pairs*, *Faces Alone* baseline and *Contour*. Each condition embraced four blocks with four trials each. Hence, we presented 16 *Face-Occupation Pairs*, 16 *Faces Alone* and 16 times the *Contour*. According to this scheme, 32 of the 48 experimental unfamiliar portraits were presented during the encoding task. The remaining 16 portraits were later used for the unconscious-conscious retrieval interaction test in the *Novel Faces* condition (see next section).

#### Test of an unconscious-conscious interaction during retrieval

In the test of interaction during retrieval, participants had to categorize celebrities with respect to their occupation—actor or politician (Fig 1B). This test encompassed four conditions: *Congruent*, *Incongruent*, *Old Face* and *Novel Face*. Each condition embraced four blocks of four trials. Sixty-four portraits of famous actors and politicians were presented as targets. The presentation of a portrait was preceded by the brief but clearly visible presentation of one of 48 non-famous faces. Of the 48 non-famous faces, 32 had previously been shown in the subliminal encoding task. The previously presented 16 *Face-Occupation Pairs* were assigned to both the *Congruent* and the *Incongruent* condition. The previously presented *Faces Alone* were assigned to the no-interaction baseline condition of *Old Faces*. The remaining 16 faces had not been presented for encoding; they were presented in the condition of *Novel Faces* (not discussed in this paper). The apparent discrepancy between the number of non-famous faces (48: 16 associative old, 16 single old, 16 not presented for encoding) and the number of famous faces (64) is explained by the fact that each of the 16 former subliminal *Face-Occupation Pairs* was used twice, namely once in the congruent condition and once in the incongruent condition. Accordingly, 16 famous faces were preceded by a congruent associative old face, 16 by an incongruent associative old face, 16 by a non-associative old face, and 16 by a new (not previously presented) face. A trial (Fig 1B) started with the presentation of a fixation cross for 1300 ms. This was followed by a 200 ms presentation of a non-famous face. Next, a fixation cross appeared again for 500 ms (= cue-target interval). Finally, a famous face was presented for 4000 ms. Participants were asked to indicate as quickly as possible whether the famous face was an actor or a politician.

#### Test of stimulus awareness

Following the fMRI experiment, participants were asked whether they had noticed something during the attention task that they performed in the first part of the fMRI experiment. When they denied, they were further asked whether they might have perceived faces or words between or within the noise masks. A yes answer led to the exclusion of this participant’s data set. Following this inquiry, all participants were informed of the subliminal presentation paradigm. Next, we administered two objective tests of stimulus awareness. In these tests, participants’ potential awareness of subliminal stimuli was assessed based on their choice behaviour. We first applied a test of face awareness that tested for the awareness of subliminally presented individual faces. Next, we applied a test of occupation awareness that tested for the awareness of subliminally presented faces plus written occupations. Each awareness tests comprised 50 trials. A trial consisted of the 12-fold subliminal presentation of a stimulus (procedure adopted from subliminal encoding in the main experiment) followed by the forced-choice test concerning this stimulus. Hence, unlike the experiment, there was no encoding-test interval. The immediate succession of a subliminal stimulus and its test facilitates the behavioural expression of stimulus awareness. In the test of face awareness, we presented 50 subliminal unfamiliar faces, each followed by the supraliminal side-by-side presentation of the target face plus a distractor face (presentation duration: 5 s). Subjects were asked to indicate which of the two faces had just been presented subliminally. In the test of occupation awareness, half of 50 faces were presented subliminally with the written occupation “actor” and the other half with “politician”. Each subliminal face-occupation pair was followed by a forced-choice test that required participants to choose between the two occupations. Participants were given 5 s to indicate which of the two occupations was just presented subliminally. In both awareness tests, participants received direct test instructions: they were instructed to base their decisions between faces or occupations on their previous conscious perception of shapes or fragments of subliminal stimuli. Direct test instructions such as this are known to be more sensitive to conscious than unconscious perception and memory [20,21], which allows measuring stimulus awareness. On the other hand, indirect retrieval tests such as the one used in the main experiment (evaluating a famous faces), are more sensitive to unconscious processing. If subjects performed above chance (binomial test; *p* <. 2) in either of these two awareness tests, their experimental data were excluded from analysis.

#### Test of knowledge of celebrities

Because our participants were young and unexperienced, we needed to ensure that they knew the politicians and actors used in the second part of the fMRI experiment. An interaction between unconscious and conscious retrieval could only occur if participants were able to identify our portraits of celebrities. To this end, participants were instructed at the end of the session to classify the previously used portraits of celebrities according to “politician” and “actor” and to retrieve the celebrities’ names. If participants claimed to know a celebrity but failed to retrieve his name, they described the celebrity and/or where they knew them from to prove identification. All participants but two were able to identify the celebrities. The experimental data of those two participants who failed were excluded from analysis.

### MRI data acquisition

Anatomical and functional images were acquired with a 3T Siemens Magnetom Trio whole-body scanner. Anatomical T1-weighted image acquisition followed a 3D-gradient echo-sequence with a spatial resolution of 1 × 1 × 1 mm^3^ (acquisition matrix = 256 × 256 voxels, 176 sagittal slices; time of repetition (TR) = 7.92 ms; echo time (TE) = 2.48 ms; flip angle (FA) = 16°; field of view (FOV) = 256 × 256 mm^2^). Structural image acquisition was carried out during the awareness tests.

Functional T2*-weighted images were acquired using a blood-oxygen-level-dependent (BOLD) sensitive, interleaved 2D-gradient echo planar single-shot pulse (EPI) sequence with a spatial resolution of 1.8 × 1.8 × 4 mm^3^ (acquisition matrix = 128 × 128 voxels, 34 transversal slices; TR = 4000 ms; TE = 32 ms; FA = 90°; FOV = 230 × 230 mm^2^).

### Behavioral data analysis

Choice reaction times (RT) acquired during the interaction task were analysed with IBM SPSS (version 20). Trials with RT deviating more than 2 *SD* from the individual mean were excluded. Because RTs were not normally distributed (Kolmogorov-Smirnov tests, all *p* <. 001; skewness > 0), nonparametric statistics were computed (Wilcoxon signed rank exact test). However, parametric testing yielded comparable results.

### fMRI data analysis

Preprocessing of volumes was carried out with the software SPM8 (Wellcome Department of Cognitive Neurology, London, UK). Volumes were slice-time corrected, realigned to the first volume, coregistered to the anatomical volume, normalized to the MNI T1 template and finally smoothed with an 8 mm (FWHM) isotropic Gaussian kernel.

First, we computed independent component analyses (ICA) and correlated the extracted components of brain activity with the time-course of the alternations between condition blocks in each of the two fMRI time-series, i.e., the encoding time-series and the interaction time-series. This analysis yields a model-and hypothesis-free estimate of functionally coupled brain areas that were engaged during unconscious associative encoding and retrieval. We computed group-level ICAs using the GIFT toolbox (http://mialab.mrn.org/software/gift/index.html). The optimal number of independent components was estimated according to the minimum description length criteria [22] in advance of the actual analysis, which was run with the Infomax algorithm [23]. This procedure resulted in the extraction of 17 independent components for the encoding time-series and 19 independent components for the interaction time-series. We were interested in components reflecting unconscious relational memory processes that covary with the occurrence of associative condition blocks. Independent components were thus sorted with respect to their regression fit with the modelled time course of associative condition blocks. Associative condition blocks contained *Face-Occupation Pairs* at encoding and *Congruent* and *Incongruent Faces* at test. False discovery rate (FDR)-corrected [24] one-sample t-tests were computed on the β-weights of sorted components to determine whether a component was significantly associated with a time-series. Significant components were subsequently checked for a-priori regions of interest, namely hippocampus and lateral- and polar temporal neocortices. Significant components containing a-priori regions of interest where then tested for a regression fit with their baseline condition (i.e., *Faces Alone* at encoding; *Old Faces* at test) to ensure that functional coupling of these components was specific for unconscious associative memory processes. Hence, a non-significant regression fit with baseline conditions was expected. Cluster statistics were calculated in SPM8 with a height threshold of *p* = .05 (family-wise-error corrected). Component images were thresholded at *Z* > 2 for visualisation. Labelling and visual inspection of the activation patterns was carried out with xjView8 (http://www.alivelearn.net/xjview8/).

While ICA is able to uncover global-scale networks, it only allows to plot the strength of association of single voxels with these networks, but is limited in providing insight into how local neural groups relate to behaviour directly. Therefore, we regressed retrieval performance (reaction time differences) onto fMRI contrasts to reveal signal changes that relate linearly to the behavioural evidence of unconscious-conscious retrieval interactions. SPM8 was used for first and second level analyses of contrasts between conditions. In the first level analysis, the time-series of each participant were modelled with a box car function convolved with a canonical hemodynamic response function. In the second level analysis, group level statistics were computed on first level contrasts using within-subject one-way ANOVAs. We entered the RT-differences recorded at test as a covariate of interest into the second-level GLM.

For subliminal encoding, the contrast (*Face-Occupation Pairs* > *Faces Alone* baseline) was correlated with the difference in reaction times in the incongruent versus congruent condition. This RT difference was chosen as a regressor over the RT difference of *Old Faces* baseline—*Congruent* because there is no difference between a prospectively congruent and incongruent face at the time of encoding, and because each *Face-Occupation Pair* was used in both the *Incongruent* and the *Congruent* condition.

For the interaction test, the contrast (*Congruent* > *Old Faces* baseline) was correlated with the difference in reaction times recorded in the *Congruent* versus the *Old Faces* condition. Both this RT measure and the fMRI contrast reflect facilitating interactions between unconscious and conscious associative retrieval. The fMRI data were not analysed regarding interfering interactions (*Incongruent* condition) because the behavioural data (see below) showed no evidence of interference between unconscious and conscious retrieval. No corrections for multiple comparisons were applied due to the small signals associated with unconscious processing [7,8,19]. The height threshold was *p* = .001 for the whole brain and *p* = .005 for the hippocampus, which was the a priori key region of interest. The extent threshold was four voxels. Labelling and visual inspection of the activation patterns was carried out with xjView8.

## Results

### Awareness tests

Participants were oblivious of both the fact of subliminal stimulation and the subliminal stimuli. For the analysis of data obtained in the two objective awareness tests, we took a conservative approach analysing the data of each individual using binomial testing. Participants with a performance above the upper 20%- cut-off of the chance distribution of correct responses were considered potentially aware of subliminal stimuli. Their data acquired in the fMRI experiment were therefore excluded from analysis. The 20%-cut-off corresponded to a hit rate of 56% (50% = chance level). Seven participants performed better than expected by chance either on the face or on the occupation awareness test. The remaining participants performed at chance level as individuals and as a group on both the face awareness test (49 ± 7.3% (*M* ± *SD*); one-sample *t*-test against 0.5: *t*(32) = -.583, *p* = .564) and the occupation awareness test (49 ± 7.2%; *t*(32) = -.527, *p* = .603). Hence, these remaining participants were unable to consciously detect subliminal faces or words or fragments thereof.

### Main Experiment: Behavioural performance

#### Subliminal processing and attention task

Participants were simultaneously processing two different streams of information at the unconscious and the conscious level. At the conscious level, participants engaged in the attention task. At the unconscious level, they processed subliminal faces and written occupations. We collected behavioural data on the attention task and calculated accuracy scores. Participants performed the attention task with high accuracy (hit rate = 94 ± 23.7%, *M* ± *SD*) indicating that they focused gaze at the middle of the screen and paid attention to the masked presentations during the whole stimulation sequence.

#### Interaction of unconscious with conscious retrieval of occupations

In the critical retrieval interaction test, participants responded to the presentation of portraits of famous actors and politicians by manually indicating their occupational category “actor” versus “politician”. Two participants performed poorly (46.9% and 65.6% correct) because they were not familiar with the celebrities; these two participants were excluded from data analysis. The remaining participants identified celebrities with 91 ± 10% (*M ± SD)* (*Congruent*), 93 ± 9% (*Incongruent*) and 92 ± 8% (*Old Faces*) correct responses. Because accuracy of choice did not differ between conditions (*F*(2,64) = 1.315, *p* = .276), reaction times were the dependent variable that could be modulated by the preceding unconscious retrieval processes.

**Fig 2:**
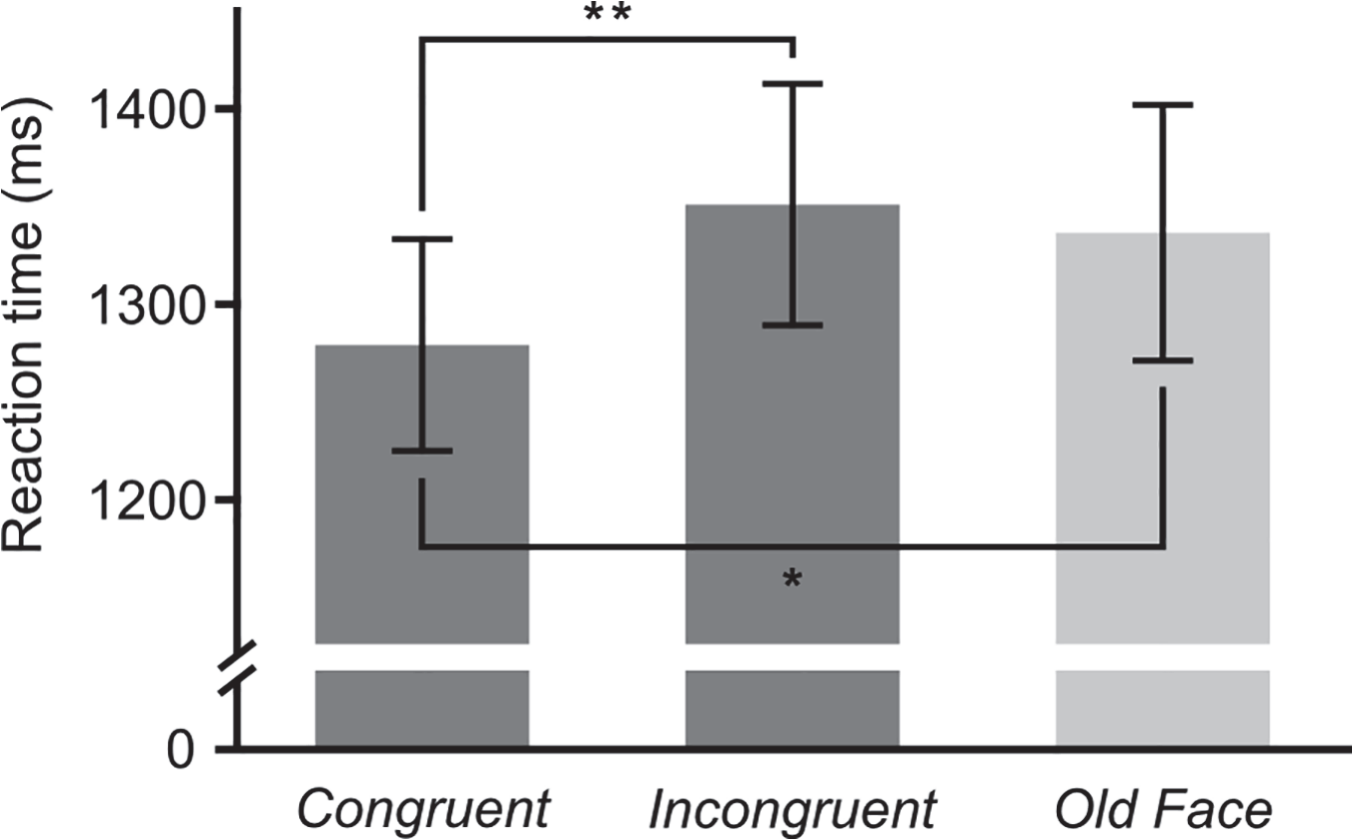
Reaction times at categorizing famous faces. Group means and SEM are displayed. *Old Face* trials (no association) are used as baseline. * Mean difference *(*Δ*M*) = 57 ms, *p* = .047, effect size *r* = .29; ** Δ*M* = 71 ms, *p* = .002, *r* = .50; one-tailed Wilcoxon signed rank exact tests.

A Wilcoxon exact test revealed a significant difference in reaction times between the congruent and the incongruent condition (*Z* = -2.850, *p* = .002, one-tailed, effect size *r* = .50) with faster responses to congruent versus incongruent famous faces. We further investigated whether this effect was due to congruence gains or incongruence costs by comparing the two conditions to the non-associative baseline condition *Old Faces*. This analysis showed that response latencies were significantly shorter to *Congruent* than *Old Faces* (*Z* = -1.689, *p* = .047, one-tailed, *r* = .29). There was no statistical difference between *Incongruent* and *Old Faces* (*Z* = -.777, *p* = .437) (Fig 2). Hence, the above behavioural effect was due to congruence gains rather than incongruence costs. The *M* ± *SD*s of the RTs in the interaction task were: 1279 ± 313 ms (*Congruent*), 1351 ± 352 ms (*Incongruent*) and 1337 ± 373 ms (*Old Faces* baseline). In conclusion, unconscious-conscious interactions were only apparent in the *Congruent* condition, where the unconsciously encoded and retrieved occupations were identical with the consciously retrieved occupations.

As all former subliminal faces in the congruent condition were also used in the incongruent condition and vice versa, a bias could arise due to their repeated presentation. This was, however, not the case: Wilcoxon exact tests showed that RTs to famous faces did not differ between the first versus second presentation of face cues in the congruent condition (*Z* = -1.099, *p* = .280, two-tailed) nor the incongruent condition (*Z* = -0.116, *p* = .916, two-tailed).

### Main Experiment: fMRI data

#### Independent component analyses

**Fig 3:**
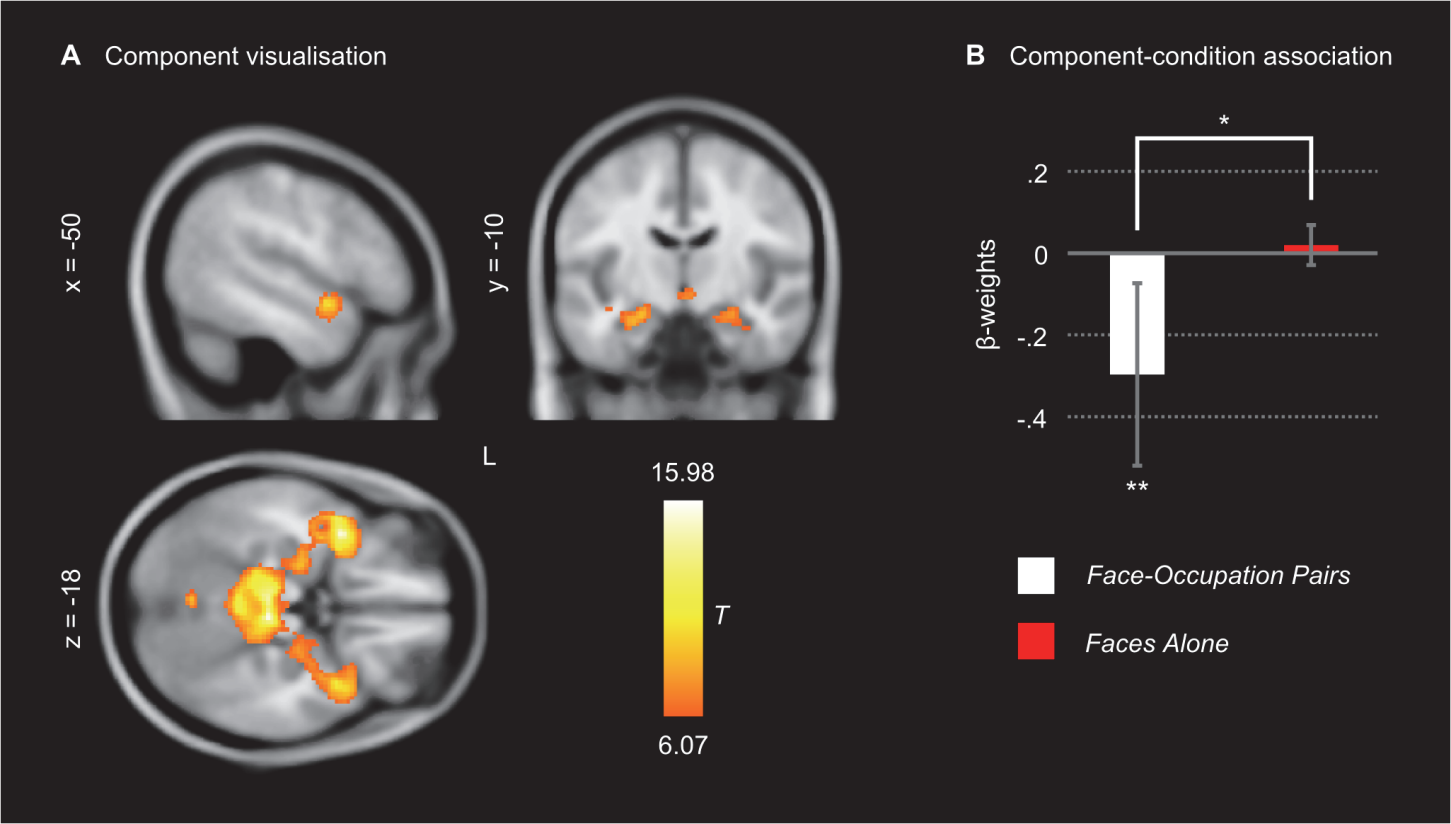
Independent component analysis (ICA) on encoding time-series. The depicted component is significantly associated with the occurrence of subliminal *Face-Occupation Pairs*. **A:** Clusters within the component encompassing bilateral hippocampus, amygdala, superior temporal sulcus, and temporal pole. These brain regions are important for episodic and semantic memory. Coordinates are according to MNI space; left is left on the coronal slice and upwards is left on the transversal slice. **B:** The temporal coupling of this network is specific for unconscious associative encoding. The component is significantly associated with the occurrence of *Faces-Occupation Pairs* (Pearson’s *r* = -.16, ** *p* = .004) but not with *Faces Alone* (Pearson’s *r* = .13, *p* = .69). The regression fit of the component is significantly better with *Face-Occupation Pairs* than with *Faces Alone* (* *p* = .044, effect size *r* = .35). The bar plot shows β-weights of the time course modelled specifically to the associative and the baseline condition. Error bars indicate the SEM.

We performed an independent component analysis (ICA) on the fMRI data acquired during the encoding fMRI time-series to explore the functional connectivity of brain regions during the subliminal processing of *Face-Occupation Pairs*. The subliminal presentation of *Face-Occupation Pairs* was associated with decreased activity in a number of functionally connected brain areas that constituted one of the obtained components (*r* = -.16, *t*(32) = -3.1, *p* = .004 < FDR critical *p* = .006) (Table 1 and Fig 3). This component included bilateral areas in the superior temporal sulcus (extending into superior and middle temporal gyrus) and temporal pole, which harbour storage sites of lexical-semantic information such as occupations [16]; bilateral hippocampus and ventromedial thalamus, required for encoding of new information [25]; and bilateral amygdala, which is considered to play an important role in face perception and evaluation. As faces convey highly significant social and emotional information, the amygdala is automatically engaged when faces are perceived [26]. Importantly, this component did not covary with the *Faces Alone* baseline (*r* = .13, *t*(32) = 0.4, *p* = .69), and the regression-fit of this component with *Face-Occupation Pairs* was significantly better than with *Faces Alone* (*t*(32) = -2.1, *p* = .044, effect size *r* = .35) (Fig 3B). In conclusion, we can assume that this component was specifically related to the semantic associative binding of subliminal faces with written occupations.

**Table 1:** Idependent component analysis of subliminal encoding: Functional network coupled with presentation of subliminal *Face-Occupation Pairs* (*r* = -.16, *p* = .004).

Region of activation	L/R	Brodmann area	X	Y	Z	N of voxels	*T*
Insula, Superior temporal sulcus (extending into Superior and Middle temporal gyrus), Temporal pole	L	21, 22, 34, 35, 38, 47	-40	10	-16	4260	15.98
*Brain Stem (multiple local maxima)*	bilat		6	-28	-20		15.79
*Hippocampus*, *Amygdala*	L		-20	-12	-16		9.12
*Hippocampus*, *Amygdala*	L		-26	-10	-20		8.58
*Entorhinal cortex*	L	28	-22	-20	-24		8.54
*Insula*	L	13	-42	-2	-4		7.17
Superior temporal sulcus (extending into superior temporal gyrus), Temporal pole	R	21, 22, 28, 34, 38, 47	40	8	-14	850	12.45
*Insula*	R	13	42	0	-6		8.73
*Hippocampus*, *Amygdala*	R		24	-12	-16		8.01
*Hippocampus*, *Amygdala*	R		30	-6	-22		7.40
*Amygdala*	R		30	-6	-16		7.35
Cerebellum, vermis	L		-4	-68	-18	29	8.80
Ventromedial thalamus	bilat		2	-12	-8	31	7.55

p <. 05 (FWE). L, left; R, right; bilat, bilateral.

The negatively (rather than positively) deflected fMRI signal during subliminal relational versus single face encoding calls for an explanation. Such negative deflections are in fact a replicable phenomenon observed during subliminal associative encoding relative to a non-associative baseline [6,7,27]. The hippocampus is active whenever an event is experienced [28] and also during rest because it retrieves and stores memories in the stream of spontaneous conscious mentation [29]. During subliminal encoding, the hippocampus may split its processing capacity between conscious spontaneous mentation and unconscious encoding of subliminal stimuli. Because backward masks interrupt the firing response of activated neurons [30,31], the processing of subliminal face-occupation pairs gets interrupted by backward masks. The more neurons are recruited to encode subliminal stimuli instead of spontaneous conscious thoughts, the more spiking activity is interrupted in the hippocampus, which reduces the fMRI signal. Relative to a relational condition, where many hippocampal neurons are recruited for unconscious encoding, a non-relational baseline condition frees hippocampal neurons from subliminal processing and makes them available for encoding the stream of spontaneous conscious thoughts. Hence, the firing of more hippocampal neurons is interrupted in the experimental than the baseline condition reducing the fMRI signal.

We also performed an ICA on the fMRI data acquired during the interaction time-series. This ICA yielded no significant component. Because all experimental conditions included the conscious inspection of famous faces and the conscious retrieval of their occupations—i.e., tasks associated with strong signal changes in the brain—, we suspect that the superimposed signal changes associated with unconscious processes were too weak and sparse for a clear modulation of the global signal.

#### Correlation of fMRI data with behavioural performance

**Fig 4:**
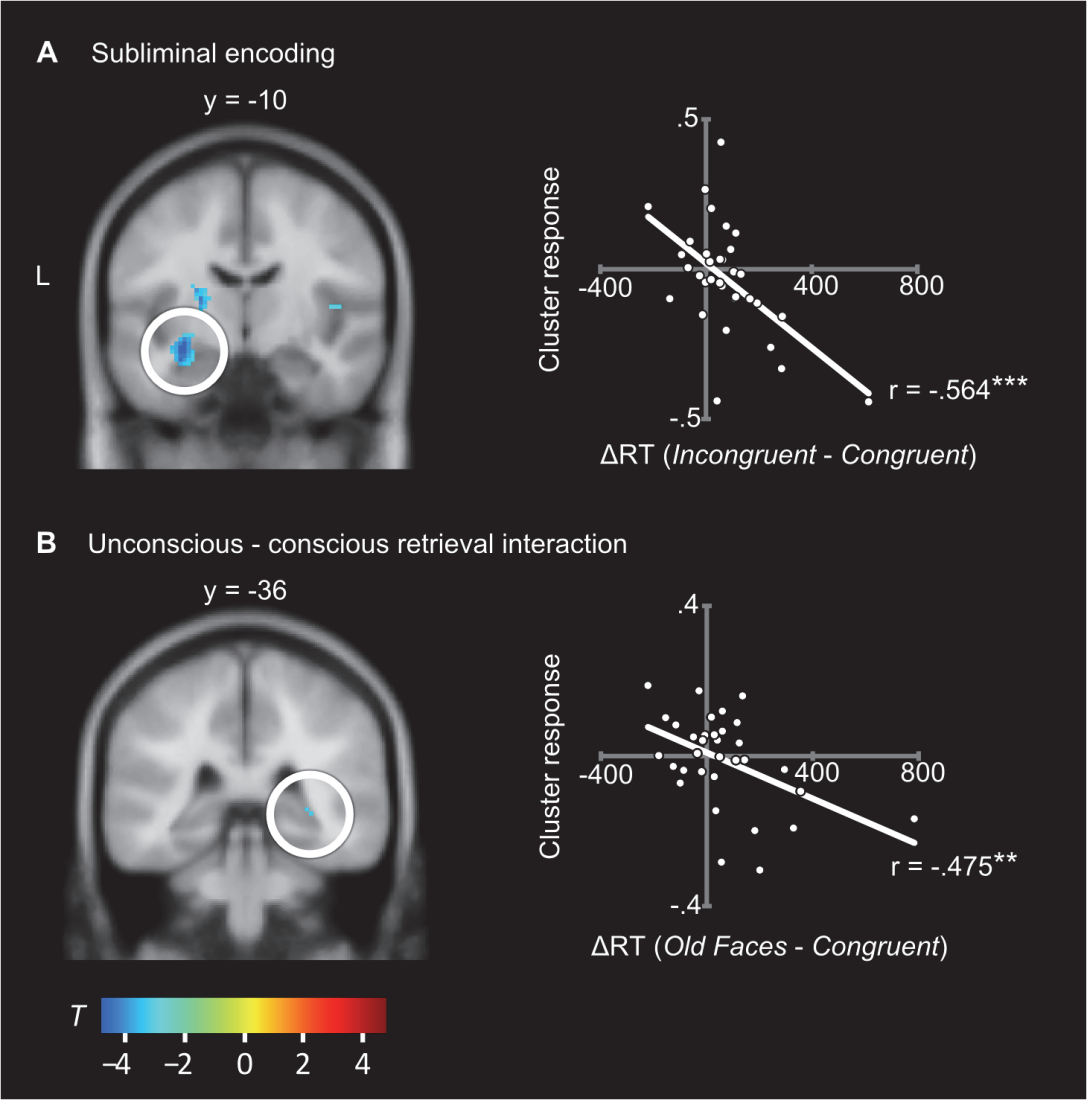
Hippocampal activity relates to retrieval performance. Locations of significant correlations are displayed on the left side of the figure. The circled hippocampal clusters correspond to the respective scatterplots displayed on the right side of the figure. Pearson-correlation coefficients are included. **A:** Correlation of the encoding contrast (*Face-Occupation Pairs* > *Faces Alone*) with the reaction time difference (ΔRT) between the *Incongruent* and *Congruent* condition. **B:** Correlation of the interaction contrast (*Congruent* > *Old Faces*) with the ΔRT between the *Congruent* and the *Old Faces* condition. Coordinates are in MNI space; the left side of the image corresponds to the left side of the brain. Warm colours indicate positive correlations (none present), cold colours negative correlations. ** *p* <. 01, *** *p* <. 001.

The reaction time difference *Incongruent*—*Congruent* was regressed onto the subliminal encoding contrast (*Face-Occupation Pairs* > *Faces Alone*) to reveal brain activation underlying successful unconscious associative encoding (Table 2). Activity reductions during subliminal associative encoding were related to faster responses at test in the congruent versus incongruent condition. Significant correlations were located in a large area that included the left hippocampus and amygdala (*r* = -.564; Fig 4A) corroborating the results of the subliminal encoding ICA, and extending them by linking brain activity at encoding with behavioural facilitation at test. Further inverse correlations were located in the right frontal operculum/insula (BA 44/13) and bilateral lentiform nuclei, i.e., putamen and globus pallidus. Activity in or near these two regions has been shown to be associated with word reading [32], more consistently within the left, rather than the right hemisphere [33]. It should be noted that unconscious information processing is often strongly supported by the right hemisphere, especially if the encoded information is emotionally relevant [34]. Hence, unconsciousness of processing might explain why we found a right hemisphere focus of activation in areas usually displaying left hemisphere dominance.

**Table 2:** Subliminal encoding-related fMRI signal correlates with behavioural facilitation during the interaction test.

Region of activation	L/R	Brodmann area	X	Y	Z	N of voxels	*T*	*r*_*cluster*_
*Negative correlation*: *Faces Alone > Face-Occupation Pairs × ΔRT(Incongruent—Congruent)*								
Lentiform nucleus	L		-22	2	2	27	4.14	-.589
Hippocampus *	L		-32	-10	-14	147	3.92	-.564
*Amygdala*	L		-26	-2	-20			
Lentiform nucleus	R		24	2	-2	20	3.77	-.582
Insula, frontal operculum	R	13 / 44	46	-4	12	8	3.60	-.545
*Positive correlation*: *Face-Occupation Pairs > Faces Alone × ΔRT(Incongruent—Congruent)*								
No Suprathreshold clusters								

p <. 001 (unc.);

*p <. 005 (unc.).

L, left; R, right; ΔRT, reaction time difference

We also regressed reaction time differences at test onto brain activity underlying the interaction of unconscious with conscious associative retrieval. Because the behavioural data indicated that the unconscious-conscious retrieval interaction yielded only congruence gains, and no incongruence costs, we focussed on congruence effects. The reduction in response time in the *Congruent* versus *Old Face* condition was regressed onto the fMRI contrast (*Congruent* > *Old Faces*). Significant negative correlations (Table 3) were located in the right posterior hippocampus (*r* = -.475; Fig 4B), the left temporal pole (BA 38) (*r* = -.572), and within the posterior end of the right superior temporal sulcus extending into the angular region (BA 22 & 39) (*r* = -.556). The posterior superior temporal sulcus is a face-responsive region often associated with theory of mind [26,35,36], and the temporal poles have been shown to specifically account for person-related semantics such as occupations [17]. That activity in these areas predicts the magnitude of the congruence effect suggests that these areas supported the unconscious recognition of the former subliminal faces and the unconscious reactivation of associated knowledge (occupations), which could then facilitate the conscious retrieval of the famous individual’s occupations. We will discuss below why this correlation was negative and what its theoretical implications are.

**Table 3:** Retrieval-related fMRI signal correlates with behavioural facilitation during the interaction test.

Region of activation	L/R	Brodmann area	X	Y	Z	N of voxels	*T*	*r*_*cluster*_
*Negative correlation*: *Old Faces > Congruent × ΔRT(Old Faces—Congruent)*								
Temporal pole	L	38	-42	20	-26	4	3.87	-.572
Superior temporal s / angular g	R	39 / 22	44	-56	14	4	3.76	-.556
Precuneus / Calcarine sulcus	R	31 / 18	2	-72	18	22	3.71	-.583
Hippocampus *	R		32	-36	0	5	2.87	-.475
*Positive correlation*: *Congruent > Faces Alone × ΔRT(Old Faces—Congruent)*								
No suprathreshold clusters								

p <. 001 (unc.);

*p <. 005 (unc.);

L, left; R, right; ΔRT, reaction time difference; s, sulcus; g, gyrus.

## Discussion

Based on previous evidence that experienced episodes can be encoded with and without consciousness and recruit the hippocampal anterior-thalamic axis and related cortices in both cases [5–8], we hypothesized that consciously and unconsciously acquired relational memories are harboured within a single, cohesive hippocampal-neocortical memory space, where they interact with each other. The reactivation of an unconsciously acquired relational memory facilitated the subsequent conscious retrieval of a semantically congruent relational memory. This facilitation was reflected in shortened reaction times and simultaneously recorded reductions in neural activation within hippocampus and neocortical storage sites thought to harbour lexical-semantic and person identity information. In the following, we discuss potential mechanisms that may underlie this facilitative unconscious-conscious retrieval interaction.

Our results point to facilitatory unconscious-conscious retrieval interactions in the congruent condition of our fMRI experiment. The supraliminal presentation of a non-famous person, who was previously presented subliminally with an occupation (e.g., politician), led to reduced response times to celebrities that share this occupation (e.g., also politician). The shortening of response times indicates that the celebrities’ occupations were preactivated by the presentation of the former subliminal faces. Accordingly, subliminal face-occupation combinations must have been encoded and stored in the first place. This finding replicates previous demonstrations of the feasibility of subliminal semantic paired-associative encoding and long-term storage using face-occupation combinations [6,7,19,37] and word pairs [8,38–40].

**Fig 5:**
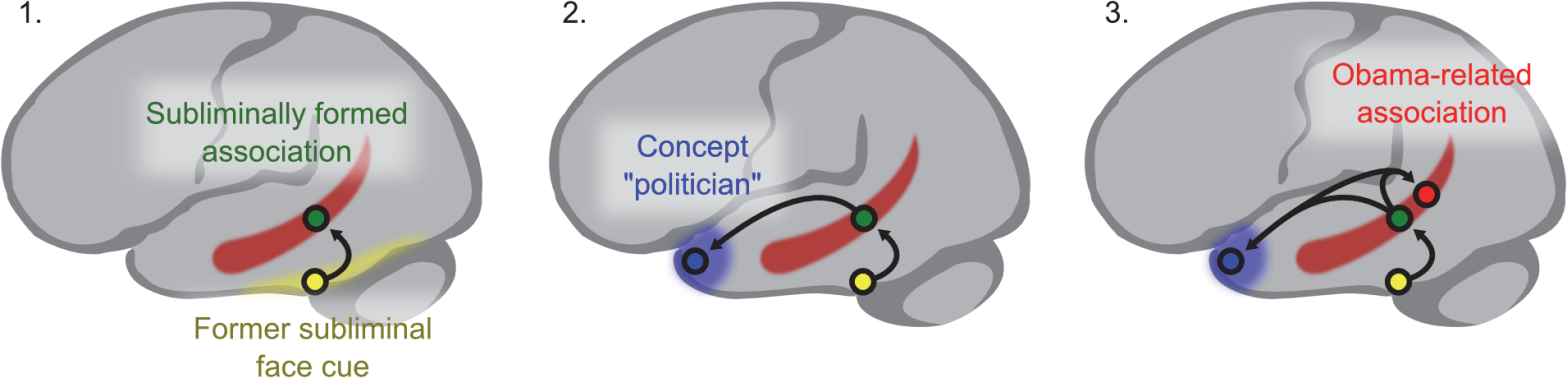
Network model of the assumed unconscious-conscious retrieval interaction. We suggest an intrahippocampal interaction mechanism as cause for the congruence effects: the supraliminal presentation of the former subliminal face elicits unconscious face recognition activating the fusiform gyrus. The fusiform signal triggers the hippocampal reactivation of the face-associated occupation (e.g., politician) (**1**), which in turn activates occupational knowledge (politician) in the lateral temporal lobe (**2**). The activated hippocampal relational engram coactivates other overlapping engrams; e.g., memories of other politicians. This intra-hippocampal preactivation facilitates the retrieval of a celebrity’s occupation (**3**). If a portrait of Obama were presented in the congruent condition, hippocampal and lateral temporal activity would be reduced compared to the baseline condition, where a hippocampal ab-initio activation would build up. Incongruence costs are not to be expected because the preceding hippocampal retrieval of a professional with another occupation (actor) would leave non-overlapping politician-related hippocampal memories unaffected. This scenario would support the view that consciously and unconsciously acquired memories are organized in a single, cohesive hippocampal-neocortical memory space with memories organized relative to their contents. Overlapping memories are linked, which supports pattern completion, abstraction and anticipation.

Savings in response times in the congruent condition went along with modulations of neural activation within hippocampus, temporal pole, superior temporal sulcus, angular gyrus, and precuneus. These neural effects were probably due to conscious rather than unconscious retrieval processes because signals associated with conscious versus unconscious mental processes are much stronger [41–43]. We suggest two possible mechanisms that may have caused response time and neural savings in the congruent condition (Fig 5).

1) Facilitation in the congruent condition may have occurred through conceptual priming of occupational knowledge stored in the lateral temporal lobe. Following the presentation of the former subliminal face and identification through face recognition units in the fusiform face area, the face-associated occupation (e.g., politician) was retrieved through hippocampal processes (Fig 5.1.) that in turn activated occupation-relevant storage sites in the lateral temporal lobe (Fig 5.2.). This preactivation of occupational knowledge sites in the lateral temporal lobe may then have primed the conscious retrieval of the famous person’s occupation (e.g., politician) reducing net activation in the lateral temporal lobe through repetition suppression due to neural sharpening or facilitation [44,45]. The reduced activation in hippocampus can be explained in terms of a sparse hippocampal recruitment for recovering the preactivated occupation of the famous face. The conceptual preactivation curtailed any unnecessary hippocampal search processes, which were necessary in the baseline condition increasing the hippocampal signal. Because the congruent condition was contrasted with the baseline condition, the activation level was relatively reduced. This mechanistic explanation of the unconscious-conscious interaction is, however, flawed by the absence of an inhibitory unconscious-conscious interaction in the incongruent condition. If conceptual priming was the crucial mechanism, we should have observed negative priming [46] in the incongruent condition (i.e. slower reactions). Because no incongruence costs occurred in the incongruent condition, conceptual priming is probably not the only mechanism underlying the unconscious-conscious interaction. A further reason why conceptual priming is unlikely to be the only mechanism at work is evidence in amnesic patients that the hippocampus is necessary for the relational encoding and retrieval of subliminal item pairs [4]. Accordingly, it can be assumed that both neocortex and hippocampus were involved in the unconscious and conscious retrieval of face-occupation associations.

2) Another mechanism seems therefore more likely, which assumes an intrahippocampal interaction as an additional cause for the congruence effects. According to this scenario, the supraliminal presentation of the former subliminal face elicited unconscious face recognition activating the fusiform gyrus. The fusiform signal triggered the hippocampal reactivation of the face-associated occupation (e.g., politician) (Fig 5.1.), which in turn activated occupational knowledge (politician) in the lateral temporal lobe (Fig 5.2.). The activated hippocampal relational engram coactivated other overlapping engrams [47], e.g., memories of other politicians. This intra-hippocampal preactivation facilitated the retrieval of the presented celebrity’s occupation (Fig 5.3.). This second scenario is in line with known characteristics of the hippocampal memory system: the hippocampal memory system forms relational networks of memory traces that share aspects. This organizational structure permits an activated memory trace to trigger the activation of memory traces that share aspects and hence overlap [47]. E.g., the presentation of Obama’s portrait in the congruent condition would be accompanied by reduced activity in the hippocampus and lateral temporal lobe due to the semantic overlap of unconscious and conscious memory traces. The preactivation of the overlapping neural populations in hippocampus during unconscious retrieval allows for a more sparing activation during conscious retrieval. In the baseline condition, no unconscious relational memories are formed that could be reactivated at test. Thus, in the baseline condition the hippocampal search process builds up fully. Incongruence costs are not to be expected because the preceding hippocampal retrieval of, say, an actor would leave non-overlapping politician-related hippocampal memories unaffected. This scenario is analogous to retrieval-induced forgetting, where a partial retrieval of information can impair the subsequent retrieval of the remaining information, if the remembered and forgotten information comes from the same semantic category [48]. This second scenario is also likely in view of earlier findings of a hippocampal role in unconscious relational encoding/retrieval [6]. If this interpretation is correct, the finding suggests that consciously and unconsciously acquired memories are organized in a single, cohesive hippocampal-neocortical memory space. There is evidence that memories are organized topologically within the hippocampus relative to their contents, with more closely related engrams represented increasingly overlapping neural populations [49]. Linked overlapping memories support pattern completion, abstraction and anticipation [47] and newly encoded information is readily integrated into pre-existing relational networks [50]. The degree of representation from consciously accessible to inaccessible memories is presumably orthogonal to the content-based organization of hippocampal memories [4].

A synergistic unconscious-conscious interaction may be counterintuitive when considering previous reports of competing interactions between implicit and explicit memories [51,52]. In these earlier studies, however, declarative memory was compared to either procedural memory or priming, managed by hippocampus, basal ganglia and neocortex, respectively. Consequently, competing memory interactions may have occurred because unconscious and conscious learning mechanisms did not share the same memory system. Conversely, the interaction in the current study was harmonious because both unconscious and conscious relational memories were supported by the hippocampus.

The current study design has its limitations. It does not allow the isolation of neural activity underlying unconscious versus conscious retrieval because the rapid succession (500 ms) of the non-famous face cue for unconscious reactivation and the famous face cue for conscious retrieval results in a blurring of signals. Therefore, we can only speculate about the mechanisms underlying the facilitatory unconscious-conscious interactions. A further limitation is that our portraits of celebrities might tap semantic information [14] besides episodic memories. Hence, the probed memory system cannot be determined beyond doubt. Yet, during the test of knowledge of celebrities it became clear that our participants were not overly familiar with many of the used famous faces and had to draw on their episodic memory. Furthermore, if semantic person knowledge was sufficient to recall occupations, unconscious-conscious interactions would likely not have modulated hippocampal signals but neocortical signals alone [14].

The classic view of the hippocampal memory system holds that consciousness is required for episodic memory formation [1–3]. However, the unconscious-conscious retrieval interaction reported here suggests that conceptually overlapping unconscious and conscious memories are stored in close association within hippocampus. An intertwined store of consciously accessible and consciously inaccessible relational hippocampal memories is compatible with the processing based memory model [5]. Also, a single, cohesive hippocampal memory space for any level of representation—unconscious to conscious—is evolutionarily sensible. As pointed out earlier, episodic memories may shift from a conscious to an unconscious representation and vice versa over time [9–11]. In both these cases, a cohesive memory space provides for a stable organizational structure of hippocampal memories. Such representational shifts appear difficult if one assumes a strict division between memory systems based on conscious access. It is more economical to assume one hippocampal memory system that serves one computational goal, namely rapidly establishing new flexible associations, irrespective of conscious access [5].
